# A Robust Recovery of Ni From Laterite Ore Promoted by Sodium Thiosulfate Through Hydrogen-Thermal Reduction

**DOI:** 10.3389/fchem.2021.704012

**Published:** 2021-06-25

**Authors:** Shoujun Liu, Chao Yang, Song Yang, Zhongliang Yu, Zhao Wang, Kang Yan, Jin Li, Xingyang Liu

**Affiliations:** ^1^ College of Chemistry and Chemical Engineering, Taiyuan University of Technology, Taiyuan, China; ^2^ Shanxi Engineering Center of Civil Clean Fuel, Taiyuan University of Technology, Taiyuan, China; ^3^ Key Laboratory for Coal Science and Technology of Ministry of Education and Shanxi Province, Taiyuan University of Technology, Taiyuan, China; ^4^ School of Chemistry and Environmental Science, Shangrao Normal University, Shangrao, China; ^5^ Taiyuan Green Coke Energy Co. Ltd., Taiyuan, China

**Keywords:** laterite ore, sodium thiosulfate, hydrogen reduction, ferronickel, magnetic separation, mechanism

## Abstract

Laterite ore is one of the important sources of nickel (Ni). However, it is difficult to liberate Ni from ore structure during reduction roasting. This paper provided an effective way for a robust recovery of Ni from laterite ore by H_2_ reduction using sodium thiosulfate (Na_2_S_2_O_3_) as a promoter. . It was found that a Ni content of 9.97% and a Ni recovery of 99.24% were achieved with 20 wt% Na_2_S_2_O_3_ at 1,100°C. The promoting mechanism of Na_2_S_2_O_3_ in laterite ore reduction by H_2_ was also investigated. The thermogravimetric results suggested the formation of Na_2_Mg_2_SiO_7_, Na_2_SO_3_, Na_2_SO_4_, and S during the pyrolysis of laterite with Na_2_S_2_O_3_, among which the alkali metal salts could destroy the structures of nickel-bearing silicate minerals and hence release Ni, while S could participate in the formation of the low-melting-point eutectic phase of FeS-Fe. The formation of low-melting-point phases were further verified by the morphology analysis, which could improve the aggregation of Ni-Fe particles due to the capillary forces of FeS-Fe as well as the enhanced element migration by the liquid phase of sodium silicates during reduction.

## Introduction

Nickel, as a ferromagnetic metal with a high corrosion resistance, plasticity, and magnetism, has been intensively used in many applications involving nickel-based alloys and stainless steel as well as in fuel cells (Sudagar et al., 2013). Stainless steel production accounts for 65% of the global nickel consumption (Moskalyk and Alfantazi, 2002). Sulfide ore and nickel laterite ore are the two main commercial nickel resources. With the gradual decline of sulfide ores deposits, nickel laterite ores are playing more important roles than before with the growth of worldwide nickel output (Quast et al., 2015a). Therefore, it is great meaningful to recover Ni effectively from nickel laterite ores from the viewpoint of sustainable development.

Nickel laterite ores can be divided into two different types, namely saprolitic and limonitic ores, based on the chemical and physical characteristics (Hidayat et al., 2008). As reported Katzagiannakis et al. (2014); Khataee et al. (2015), the laterite cannot be easily used through physical methods because of its poor crystallinity and fine grains. Zhu et al. (2012a) investigated the mineralogy and crystal chemistry of a low-grade limonite-type nickel laterite ore and found that the Ni was mainly present in the goethite and silicates phases. Therefore, it is required to eliminate the restriction of the goethite and silicates phases first for the Ni liberation, which can then be beneficiated by subsequent treatment technology, such as reduction roasting followed by magnetic separation (Zhu et al., 2012b).

Recently, several researchers have focused on the reduction roasting of laterite ore with different reductants such as carbon, CO or H_2_, followed by magnetic separation (Lu et al., 2013; Quast et al., 2015b). In addition, compared with C and CO, hydrogen has a good carbon footprint, and the carbon footprint is relatively stable, close to zero, which is conducive to better carbon neutralization. (Valente et al., 2020). In recent years, a number of studies (Hu et al., 2019; Zhang et al., 2020) have focused on the selective conversion of biomass into renewable energy, such as hydrogen, As a reducing agent, hydrogen has strong environmental benefits. Moreover, the additives, such as Na_2_CO_3_, S, NaCl, and Na_2_SO_4_, have been used to enhance the enrichment ratio of Ni (Li et al., 2012; Jiang et al., 2013). Oliveira V D A et al. (de Alvarenga Oliveira et al., 2020) studied the reduction kinetics of limonite under the action of hydrogen and found that the diffusion of reagent (H_2_) or product (gaseous H_2_O) through the ash layer is a slow process step. Harris et al. (2011) selected sulfur (S_2_) as the additive for the laterite ores calcination at 450, 500, and 550°C and observed that the nickel extraction and selectivity were improved by the increasing temperature. Li et al. (2012) studied the beneficiation of nickeliferous laterite by reduction roasting in the presence of sodium sulfate and noticed when the laterite was mixed with sodium sulfate and then reduced at 1,100°C for 60 min, the Ni grade of the concentrate and recovery were increased to 9.48 and 83.01%, respectively. Lu et al. (2013) investigated the effect of sodium sulfate on the hydrogen reduction process of nickel laterite ore. In conclusion, the alkali metal additives can be used to improve the reactivity of reducing agents, and to break the structure of Ni-containing silicate. While the sulfur additives are usually applied to improve the grade of Ni. Based on the discuss above, it is more excepted to find an green and cheap additive that could play the dual roles of alkali metals and sulfur compounds at the same time, because this could not only remarkably decrease the operation cost, but also alleviate the environmental pollution.

Sodium thiosulfate is a common raw chemical material that can provide alkali metals and elemental sulfur simultaneously. Mcamish and Johnston (1976) analyzed the sulfur exchange and decomposition kinetics in solid Na_2_S_2_O_3_ and found that the decomposition of Na_2_S_2_O_3_ occurred in a temperature range from 290 to 350°C, with the main products of S, Na_2_S and Na_2_SO_4_. However, limited studies on this topic have been reported, especially the effect and mechanism of sodium thiosulfate on promoting the laterite ore reduction.

Therefore, in current study, sodium thiosulfate was employed to improve the grade and recovery of nickel by hydrogen reduction of laterite followed by magnetic separation. The effects of temperature, reduction time, and sodium thiosulfate on the nickel laterite reduction were investigated. The samples were characterized in detail by inductively coupled plasma-atomic emission spectrometry (ICP-AES), X-ray diffraction analysis (XRD), and scanning electron microscopy coupled with an energy dispersive X-ray spectroscopy (SEM-EDS). Based on the collected data, the promotion mechanism of sodium thiosulfate for hydrogen-thermal reduction of laterite ore was proposed.

## Experimental

### Materials

Nickel laterite ore from Indonesia was used as the raw ore, which is Limonitic Nickel Ore. Reagent grade sodium thiosulfate (Na_2_S_2_O_3_), purchased from Tianjin Kemiou Chemical Reagent Co., Ltd., China, was selected as the additive. Hydrogen (99.99 vol%) was employed as the gaseous reductant. Nitrogen (99.99 vol%) was employed as the protective and balance gas. All the gases were supplied by Taiyuan iron and steel Co., Ltd.

### Sample Preparation

The samples were prepared as follows. First, the as-received nickel laterite ore was air-dried at 105°C overnight in an oven. Second, the dried nickel laterite ore was crushed to a particle size of less than 2 mm using a jaw crusher. After, the crushed nickel laterite ore was ground using a laboratory-scale ball mill and was subsequently sieved to a particle size of less than 80 mesh. Finally, the raw ore and Na_2_S_2_O_3_ are ground with a sample maker, then sieved with a 100–140 mesh sieve, and finally mixed in a beaker, the Na_2_S_2_O_3_ contents of 5, 10, 15, 20 or 25 wt%. The corresponding nickel laterite ores with different contents of sodium thiosulfate were denoted as prepared ore (PO).

### Reduction Experiments and Magnetic Separation Process

A typical reduction experiment of PO was conducted as follows. Approximately 80 g of PO loaded into a stirred fixed-bed reactor (Lu et al., 2013). The temperature of the PO during roasting and reduction roasting with H_2_ was monitored by a thermocouple inserted into the reactor. Then PO was heated at 450°C for 1 h under a nitrogen atmosphere (0.7 L/min). Thereafter, the reactor was heated to the final reaction temperature, and then 27 L/min of H_2_/N_2_ (45/55) was introduced to reduce the ores. After the reduction process was complete, the ore samples were cooled to room temperature under a nitrogen atmosphere (0.1 L/min). The effects of the reduction temperature, time, and the dosage of Na_2_S_2_O_3_ were investigated.

After reduction, 5 g of product was ground to a particle size of 90 wt% passing 0.043 mm using a rod mill. These particles were then separated in a Magnetic Tube (XCGS-73 Davies) with a magnetic field intensity of 0.1 T to obtain the Fe-Ni concentrates. The grade of Ni in sample were determined by chemical analysis of dimethylglyoxime photometry., and the grade of Fe were determined by the potassium dichromate method. The recovery rate of Ni was calculated according to the following equation (Lu et al., 2013):

$$R_{Ni/Fe}=m_{1}/m_{2}\times 100\%$$ (1)

where $R_{Ni/Fe}$ is the nickel or iron recovery rate, and $m_{1}$ and $m_{2}\;$ are the elemental nickel or iron contents in the concentrate and the reduced samples, respectively.

### Analysis and Characterization

The main chemical composition of nickel laterite ore and the contents of Fe and Ni in each sample were determined by inductively coupled plasma atomic emission spectrometry (ICP-AES-9000(N + M), a commercial product of Thermo Jarrell-Ash Corp., United States. The distribution of nickel in the laterite was analyzed by chemical phase analysis, as reported previously (Mcamish and Johnston, 1976).

The crystalline phases of the samples were recorded using an X-ray diffractometer (Rigaku D/Max 2,500, Japan) under the following conditions: Cu-Kα radiation of 150 mA and a scanning rate of 3°/min from 5 to 85°. The morphological changes were analyzed by scanning electron microscopy with an energy dispersive X-ray spectroscopy (SEM-EDS) (Phenom ProX, Netherlands).

A Setaram SETSYS TGA was used to study the weight loss during the decomposition of laterite ore blended with Na_2_S_2_O_3_. Approximately 25 mg of mixture was heated under 100 ml/min of Ar flow with a ramp rate of 10°C/min from room temperature to 1,050°C. The obtained TG data was further processed to determine the net weight loss for the decomposition analysis using the following equation:

$$W_{L}=\left(m-m_{T}\right)/\left(m-m_{1050}\right)\times 100\%$$ (2)

where $W_{L}$ is the net weight loss; $m_{T}$ is the mass of the sample at the temperature of T; $m_{1050}$ is the mass of the sample at 1050°C; and m is the initial mass of sample.

## Result and Discussion

### Characterization of Raw Nickel Laterite Ore

Table 1 shows the main chemical composition of the raw nickel laterite, which contained 1.41 wt% of Ni, 24.14 wt% of Fe, 3.15 wt% of Al_2_O_3_, 1.46 wt% of CaO, 14.58 wt% of MgO, 29.36 wt% of SiO_2_, 0.018 wt% of P_2_O_5_, 1.46 wt% of MnO_2_, and 0.065 wt% of Co. Valix and Cheung (2002) investigated the mineralogical properties of nickel laterite and found that the element content ranges of limonitic nickel ore was Ni 1.5–1.8 wt%, Fe 25–40 wt%, Co 0.02–0.1 wt%, MgO 5–15 wt%, and Cr_2_O_3_ 1-2 wt%.

**TABLE 1:** Main chemical composition of nickel laterite ore (wt%).

TFe	TNi	Al_2_O_3_	CaO	MgO	Cr_2_O_3_	SiO_2_	P_2_O_5_	MnO_2_	Co
24.14	1.41	3.15	1.46	14.58	1.08	29.36	0.018	1.46	0.065

Table II. Measured and Theoretically Calculated Mass Losses as wel.

**FIGURE 1:**
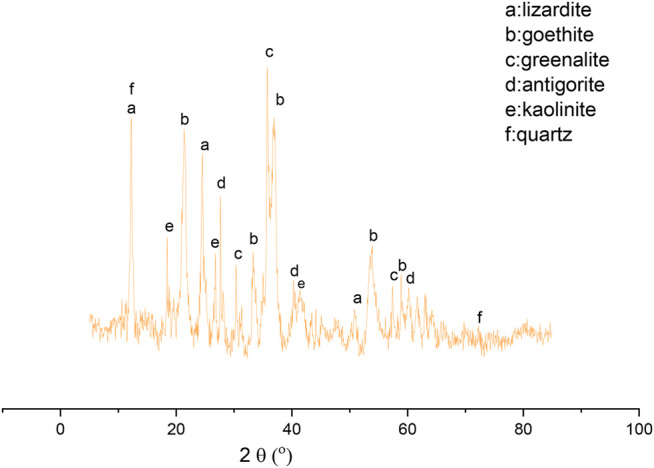
XRD pattern of the nickel laterite ore.

Figure 1 shows the XRD patterns of the raw nickel laterite ore. The raw laterite ore mainly consisted of lizardite, goethite, greenalite, antigorite, kaolinite, and quartz. However, no nickel-containing phases were evident in the XRD patterns, which may be due to the low nickel content or the poor crystallinity of the nickel phase in the raw laterite ore (Zeissink, 1969). This indicated that the nickel laterite ore used in this study was a typical transition layer laterite ore.

To understand the nickel form in the laterite ore, the nickel distribution state of the studied laterite was examined by chemical titration, as shown in Table 2. The nickel mainly existed as the silicate minerals, which accounted for 84.4% of the total nickel, followed by the oxide minerals (8.51%), the sulphides minerals (4.96%), and the adsorption minerals.

**TABLE 2:** Nickel distribution state of the studied laterite.

Type	Silicates	Oxides	Sulphides	Adsorption	Ni_total_
Contents/wt%	1.19	0.12	0.07	0.03	1.41
Ratio/%	84.40	8.51	4.96	2.13	100

Because some magnesium ions contained in the serpentine could exchange Ni ions during the weathering of laterite ore, the nickel enrichment process in serpentine olivine and other silicates ores can be expressed as follows (Kim et al., 2010):

$$\text{Mg}\;\;\text{serpentine}+Ni^{2+}\;\to \mathrm{Ni}\;\;\text{serpentine}+{\text{Mg}}^{2+}$$ (3)

Therefore, the breakage of the nickel-rich sillicate structures is the key step for improving the nickel recovery by calcination.

### Effects of Different Reduction Conditions on Ni Beneficiation

#### Effect of Sodium Thiosulfate Dosage on Ni and Fe Beneficiation

**FIGURE 2:**
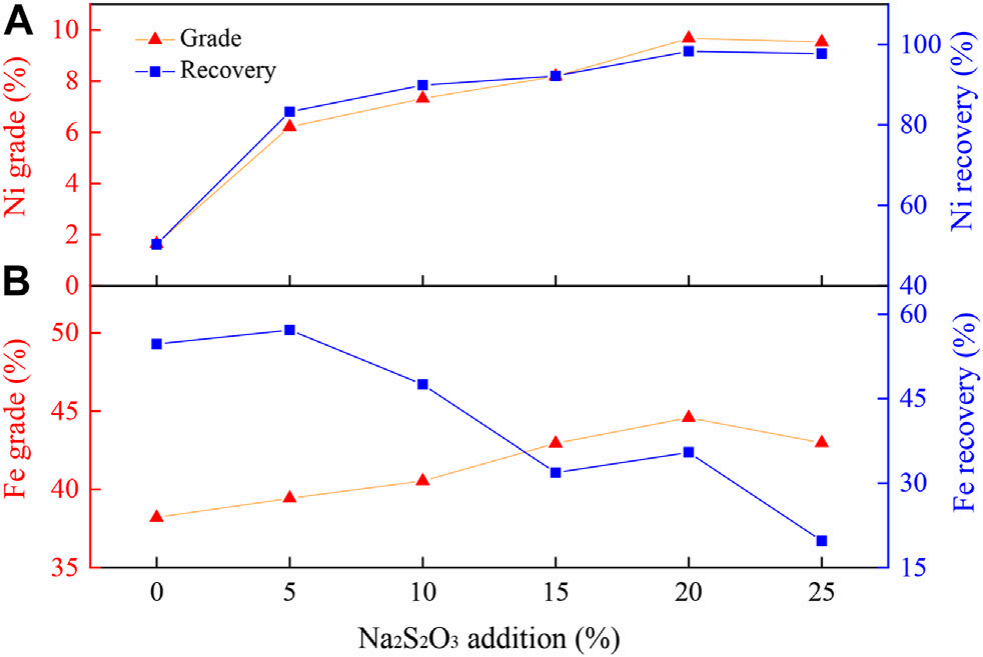
**|** Effect of Na_2_S_2_O_3_ addition on the **(A)** nickel and **(B)** iron enrichment.

The Ni grade and recovery of laterite reduced with varied dosages of Na_2_S_2_O_3_ (ranging between 0 and 25 wt%) are displayed in Figure 2. As shown in Figure 2A, without Na_2_S_2_O_3_, the nickel grade and recovery only reached 1.65 and 50.34%, respectively. However, with 25 wt% Na_2_S_2_O_3_, the nickel grade and recovery were improved to 9.53 and 97.73%, respectively. With the increase in the Na_2_S_2_O_3_ addition, the nickel grade and recovery were increased as well. However, the iron grade was only slightly improved, and the iron recovery decreased significantly with the increase in Na_2_S_2_O_3_ (Figure 2B). This shows that the addition of Na_2_S_2_O_3_ was not only conducive to the improvement of the nickel grade but also had a significant effect on the iron removal, which could then achieve the selective recovery of nickel and iron. When the addition of Na_2_S_2_O_3_ exceeded 20 wt%, both the grade and recovery of nickel and iron were only slightly changed compare to the result with the Na_2_S_2_O_3_ of 25 wt%. Hence, based on the cost and quality of the nickel concentrate, a 20 wt% addition of Na_2_S_2_O_3_ was chosen for the following experiments.

Experimental conditions: temperature, 1000°C; reduction time, 90 min; flow rate of H_2_/N_2_, 27 L/(min kg); gas volume fraction of H_2_, 45 vol%; grinding fineness, 85 wt% passing 0.074 mm; magnetic field intensity, 0.156 T.

#### Effect of Reduction Temperature on the Concentration of Ni and Fe Beneficiation

Experimental conditions: Na_2_S_2_O_3_ addition, 20 wt%; reduction time, 90 min; flow rate of H_2_/N_2_, 27 L/(min·kg); gas volume fraction of H_2_, 45 vol%; grinding fineness, 85 wt% passing 0.074 mm; magnetic field intensity, 0.156 T.

**FIGURE 3:**
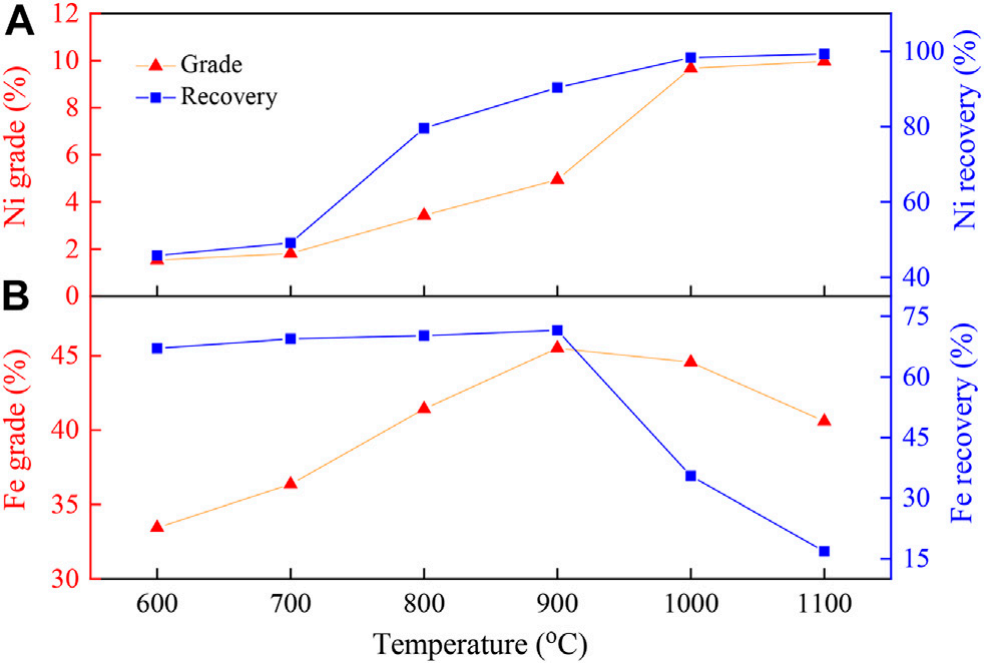
Effect of reduction temperature on **(A)** nickel and **(B)** iron enrichment.

Figure 3 shows the evolution of the magnetic products as a function of the reduction temperature. Figure 3A shows that the grade and recovery of Ni in the magnetic products increased with increasing reduction temperature, which increased from 1.53 to 45.77% at 600°C to 9.97% and 99.24% at 1100°C, respectively. Under the low reduction temperatures (below 700°C), the increase in the nickel grade and recovery of the concentrates were still not evident, which may have been because the promoting reaction between Na_2_S_2_O_3_ and laterite was not apparent. Nevertheless, when the temperature exceeded 800°C, the nickel grade and recovery were sharply improved. Thus, 800°C could be considered to be an inflection point. At 800°C, recrystallization of the amorphous silicate occurred (Lu et al., 2013). The restructuring of magnesium silicate promoted the reaction of silicate with Na_2_S_2_O_3_, and thus, the exchange between nickel and Na^+^ was accelerated. Finally, the nickel grade and recovery of concentrate ore were improved. The nickel grade and recovery could be remarkably increased by increasing the temperature after the inflection point. When the reaction temperature was 1100°C, the nickel grade and recovery reached their maximum values of 9.97 and 99.24%, respectively.

Figure 3B shows that the grade of iron was only slightly changed with the increase in temperature, while the recovery rate of iron firstly increased before 900°C and then decreased as the reduction temperature increased, which may be due to the gradually increased reduction degree of iron oxides as the temperature increased. When the temperature was further increased, Na_2_S_2_O_3_ could interact with the iron phase to form FeS substances (as shown in Figure 8) and eliminate impurities, and hence, the selective nickel-iron recovery rate could be achieved. Since these results were slightly higher than those obtained at the reaction temperature of 1000°C (9.67% of nickel grade and 98.32% of nickel recovery), the reaction temperature of 1000°C was selected for the following experiments.

#### Effect of Reducing Time on Concentration of Ni and Fe Beneficiation

**FIGURE 4:**
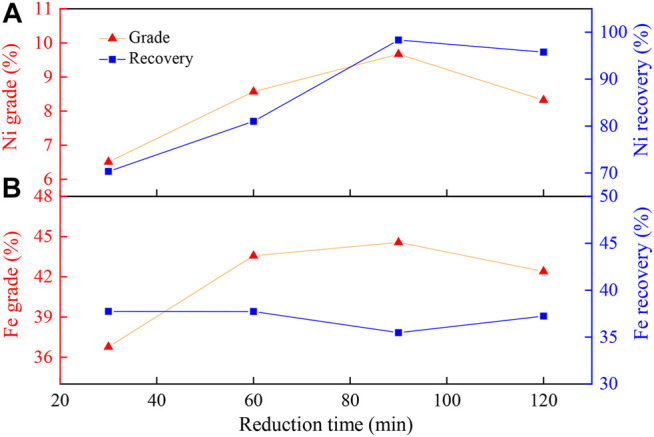
Effect of reduction time on **(A)** nickel and **(B)** iron enrichment. Experimental conditions: Na_2_S_2_O_3_ addition, 20 wt%; temperature, 1000°C; flow rate of H_2_/N_2_, 27 L/(min·kg); gas volume fraction of H_2_, 45 vol%; grinding fineness, 85 wt% passing 0.074 mm; magnetic field intensity, 0.156 T.

The magnetic separation results of the prepared ore (PO) reduced at 1,000°C as a function of reduction time ranging from 30 to 120 min are shown in Figure 4. The prolonged reduction time could significantly promote the Ni recovery, whereas it could only slightly improve the nickel grade (Figure 4A). As the reaction time increased, the reduction degree, the nickel grade, and recovery rate of Ni increased initially. However, when the reaction exceeded a certain degree, the iron oxides were also over-reduced to metal iron, which could result in the nickel grade declining in the concentrate. Figure 4B shows that the grade and recovery rate of iron remained unchanged with the increase in the reduction time, which indicated that the influence of the reduction time on the grade and recovery rate of iron was negligible.

#### Effect of H_2_ Ratio on Concentration of Ni and Fe Beneficiation

**FIGURE 5:**
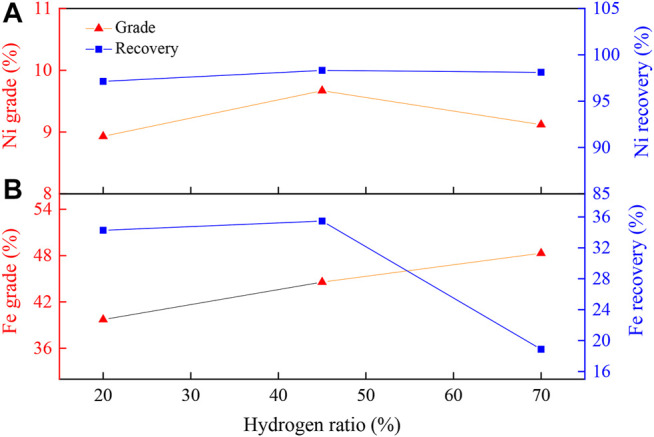
Effect of H_2_ ratio on **(A)** nickel and **(B)** iron enrichment. Experimental conditions: pre-calcination, 450°C, 60 min; temperature, 1000°C; reduction time, 90 min; flow rate of H_2_/N_2_, 27 L/(min kg); grinding fineness, 85 wt% passing 0.074 mm; magnetic field intensity, 0.156 T.

Figure 5 displays the effect of H_2_ ratio on the nickel and iron enrichment. As shown in Figure 5A, with the increase in the H_2_ ratio, the nickel grade increased first and then decreased, while its recovery rate remained almost unchanged. When the H_2_ ratio was 45 vol%, the nickel grade and recovery reached maximum values of 9.67 and 98.32%, respectively. This was attributed to the different reduction states of the iron oxides with different H_2_ ratios. With a H_2_ ratio of 20 vol%, most of the iron oxides were reduced to Fe_3_O_4_. During the magnetic separation process, due to the strong magnetic property of Fe_3_O_4_, large amounts of Fe_3_O_4_ and Ni were magnetically separated into the concentrate, leading to decline in concentrate nickel grade. With the H_2_ ratio of 70 vol%, most of the iron oxides were reduced to Fe, which would then melt with nickel to form ferronickel above 900°C (Zhu et al., 2012a), causing a high iron content in the nickel concentrate and a decreased nickel grade. At a H_2_ ratio of 45 vol%, iron oxide was reduced to non-magnetic FeO, which could be discarded with the gangue component in the subsequent magnetic separation, thereby reducing the recovery of iron and improving the nickel grade. According to Figure 5B, as the proportion of hydrogen increased, the iron grade did not change significantly, while the iron recovery rate showed a peak value at 45 vol%. The iron recovery rate could be enhanced due to the improved reduction degree of iron oxides with increasing H_2_ ratio. When the H_2_ ratio was further increased, the reduction degree of Na_2_S_2_O_3_ and iron was further increased, forming FeS, which affected the recovery rate of ferric oxygen. Therefore, a 45 vol% of H_2_ ratio was selected as the optimal hydrogen ratio.

### Reduction Mechanism of Nickel Laterite Ore Improved by Sodium Thiosulfate

#### Behavior of Pyrolysis Characteristics of Laterite With Na_2_S_2_O_3_

**FIGURE 6:**
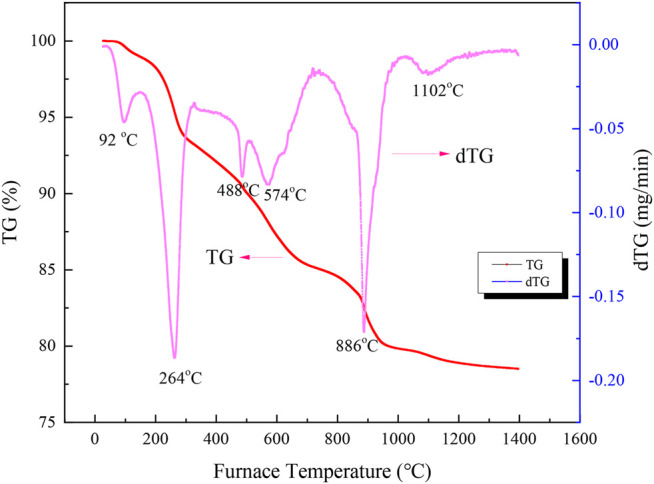
TG-DTG curves of the Na_2_S_2_O_3_/laterite blend.

To clarify the reaction mechanism of Na_2_S_2_O_3_ on the laterite nickel ore reduction, the thermal characteristics of the laterite blended with 20 wt% Na_2_S_2_O_3_ were investigated by TG-DTG, as shown in Figure 6.

From Figure 6, five steps can be found in the net weight loss curve of mixture during the heating process. The first step occurred between room temperature to 141°C, with a weight loss of 1.03%, which could be due to the free water removal of laterite nickel ore and Na_2_S_2_O_3_ (Mcamish and Johnston, 1976). The second step occurred between 152 to 298°C, and 4.87% weight loss was observed during the process, which was attributed to the bound water loss from Na_2_S_2_O_3_·5H_2_O, as follows:

$$Na_{2}S_{2}O_{3}\cdot 5H_{2}O=Na_{2}S_{2}O_{3}+5H_{2}O$$ (4)

In the temperature range from 416 to 503 °C, 1.84% of the mass was lost, which may have been attributed to the dehydroxylation of goethite-type hematite in laterite nickel ore, shown as follows (Lu et al., 2013).

$$2FeO\left(OH\right)=Fe_{2}O_{3}+H_{2}O\left(g\right)$$ (5)

The fourth stage showed a 4.24% net weight loss from 523 to 704°C, which was assigned to the decomposition of Na_2_S_2_O_3_ to S and Na_2_SO_3_ at 488°C and the dehydroxylation of serpentine and kaolinite in the laterite nickel ore with a peak positioned at 574°C (Lu et al., 2013). The sulphidation of a nickeliferous lateritic ore was studied at temperatures between 450 and 1100°C, and the nickel could be selectively sulfidized to form a nickel-iron sulfide (Harris et al., 2011; Harris et al., 2013; Ding et al., 2020). These processes can be described by the following reactions:

$$Mg_{3}{\left(OH\right)}_{4}Si_{2}O_{5}=Mg_{2}SiO_{4}+MgSiO_{3}+2H_{2}O\left(g\right)$$ (6)

$$Al_{2}{\left(OH\right)}_{4}Si_{2}O_{5}=Al_{2}O_{3}\cdot 2SiO_{2}+2H_{2}O\left(g\right)$$ (7)

$$Na_{2}S_{2}O_{3}=S\left(g\right)+Na_{2}SO_{3}$$ (8)

The final step occurred from 759 to 1042°C with a net weight loss of 5.26%. In this stage, the disproportionation of Na_2_SO_3_ to Na_2_SO_4_ and Na_2_S occurred as follows:

$$4Na_{2}SO_{3}=3Na_{2}SO_{4}+Na_{2}S$$ (9)

The recrystallization of magnesia-nickel silicate by the sodium salts also occurred after H_2_ introduction, which could transform the nickel in the silicate to form NiO, as Equation 8 (Li et al., 2012).

$$H_{2}\left(g\right)+2\left(Mg,Ni\right)SiO_{4}+Na_{2}SO_{4}=Na_{2}Mg_{2}Si_{2}O_{7}+2NiO+SO_{2}\left(g\right)+H_{2}O\left(g\right)$$ (10)

$$H_{2}\left(g\right)+Ni_{2}SiO_{4}+Na_{2}SO_{4}=Na_{2}SiO_{3}+2NiO+SO_{2}\left(g\right)+H_{2}O\left(g\right)$$ (11)

$$H_{2}\left(g\right)+2Ni_{2}SiO_{4}+Na_{2}SO_{4}=Na_{2}Si_{2}O_{5}+4NiO+SO_{2}\left(g\right)+H_{2}O\left(g\right)$$ (12)

$$2H_{2}\left(g\right)+Ni_{2}SiO_{4}+2Na_{2}SO_{4}=Na_{4}SiO_{4}+2NiO+\;2SO_{2}\left(g\right)+2H_{2}O\left(g\right)$$ (13)

$$3H_{2}\left(g\right)+2Ni_{2}SiO_{4}+3Na_{2}SO_{4}=Na_{6}Si_{2}O_{7}+4NiO+3SO_{2}\left(g\right)+3H_{2}O\left(g\right)$$ (14)

$$H_{2}\left(g\right)+NiO=Ni+H_{2}O\left(g\right)$$ (15)

$$H_{2}\left(g\right)+Ni_{2}SiO_{4}=NiSiO_{3}+Ni+H_{2}O\left(g\right)$$ (16)

$$3H_{2}\left(g\right)+2Ni_{2}SiO_{4}=\;NiSi_{2}O_{5}+3Ni+3H_{2}O\left(g\right)$$ (17)

$$H_{2}\left(g\right)+2Ni_{2}SiO_{4}=Ni_{3}Si_{2}O_{7}+Ni+H_{2}O\left(g\right)$$ (18)

**FIGURE 7:**
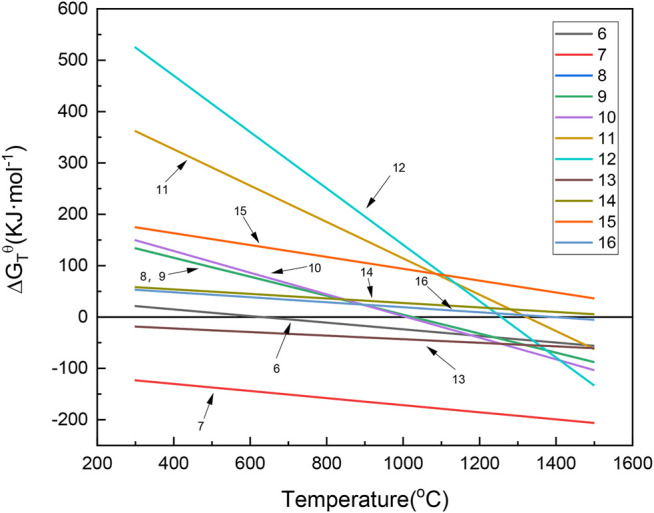
Change of the Gibbs free energy with and without the addition of sodium thiosulfate under an H_2_ atmosphere. (6): Mg_3_(OH)_4_Si_2_O_5_ = Mg_2_SiO_4_+MgSiO_3_+2H_2_O(g); (7): Al_2_(OH)_4_Si_2_O_5_ = Al_2_O_3_·2SiO_2_+2H_2_O(g); (8): Na_2_S_2_O_3_ = S(g) + Na_2_SO_3_; (9): 4Na_2_SO_3_ = 3Na_2_SO_4_+Na_2_S; (10): H_2_(g)+2(Mg,Ni)SiO_4_+Na_2_SO_4_ = Na_2_Mg_2_Si_2_O_7_+2NiO + SO_2_(g)+H_2_O(g); (11): H_2_(g) +Ni_2_SiO_4_ + Na_2_SO_4_ = Na_2_SiO_3_ + 2NiO + SO_2_(g) + H_2_O(g); (12): H_2_(g) +2Ni_2_SiO_4_ + Na_2_SO_4_ = Na_2_Si_2_O_5_ + 4NiO + SO_2_(g) + H_2_O(g); (13): 2H_2_(g) +Ni_2_SiO_4_ + 2Na_2_SO_4_ = Na_4_SiO_4_ + 2NiO + 2SO_2_(g) + 2H_2_O(g); (14): 3H_2_(g) +2Ni_2_SiO_4_+3Na_2_SO_4_ = Na_6_Si_2_O_7_ + 4NiO +3SO_2_(g) +3H_2_O(g); (15): H_2_(g)+NiO = Ni + H_2_O(g); (16): H_2_(g) +Ni_2_SiO_4_ = NiSiO_3_+Ni + H_2_O(g).

Figure 7 shows the Gibbs free energy of laterite ore reduction by H_2_ with and without the addition of sodium thiosulfate. The decomposition of Na_2_S_2_O_3_ to S and Na_2_SO_3_ occurred at about 680 K, and Na_2_SO_3_ could decompose to Na_2_SO_4_ and Na_2_S at 298 K. Eqs 8–12 are reduction reactions of laterite nickel ore with sodium sulfate under a H_2_ atmosphere. According to Supplementary Table S1 and S2, thermodynamic data suggests that the above reactions could occur at approximately 1000 K. However, laterite nickel ore cannot be reduced by H_2_ without sodium sulfate, corresponding to Eqs 13–16. Therefore, laterite nickel ore can be effectively reduced by H_2_ with the help of sodium thiosulfate.

#### Phase Transformations of Nickel Laterite Ore During Reduction

**FIGURE 8:**
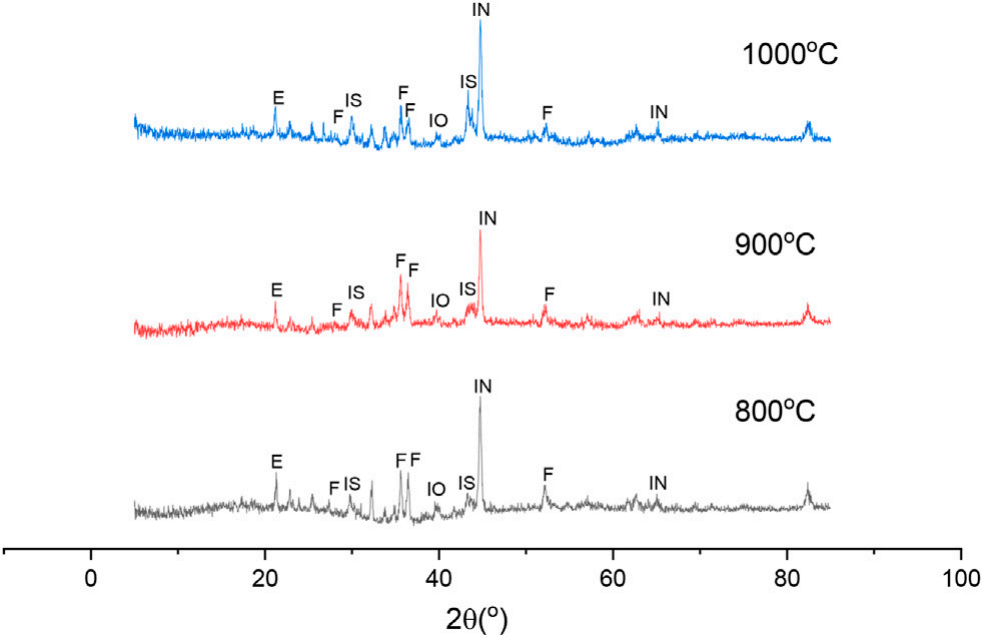
XRD patterns of roasted ores under different reduction roasting temperatures: (IN) Iron-Nickel, (IS) FeS, (F) Na_2_Mg_2_Si_2_O_8_ Forsterit, (E) Enstatite, (IO) Iron Oxide.

To reveal the effect of sodium thiosulfate on the reduction and beneficiation of nickel laterite, the XRD patterns of the reduction residue of laterite ore were analyzed, as shown in Figure 8. With the increase in the reduction temperature, the intensity of the nickel-iron increased gradually with the consumption of iron oxide, which indicates that the higher temperature was beneficial for the accumulation of nickel-iron. This accumulation may have been due to the formation of the low melting eutectic phase of the FeS-Fe system, which could improve the mobility of the reaction system. Therefore, the transfer between the phases was accelerated (Li et al., 2012; Jiang et al., 2013; Lu et al., 2013). The peak of Na_2_Mg_2_Si_2_O_8_ increased gradually with the increase in the reaction temperature, which indicated that the heating could promote the activity of the sodium salts and accelerated the reaction between the sodium salts and nickel-magnesium. Meanwhile, the nickel release and reduction were also promoted, thereby improving the grade of the nickel concentrate (de Alvarenga Oliveira et al., 2019).

#### Melting and Particle Growth of Ni-Fe During the Reduction

**FIGURE 9:**
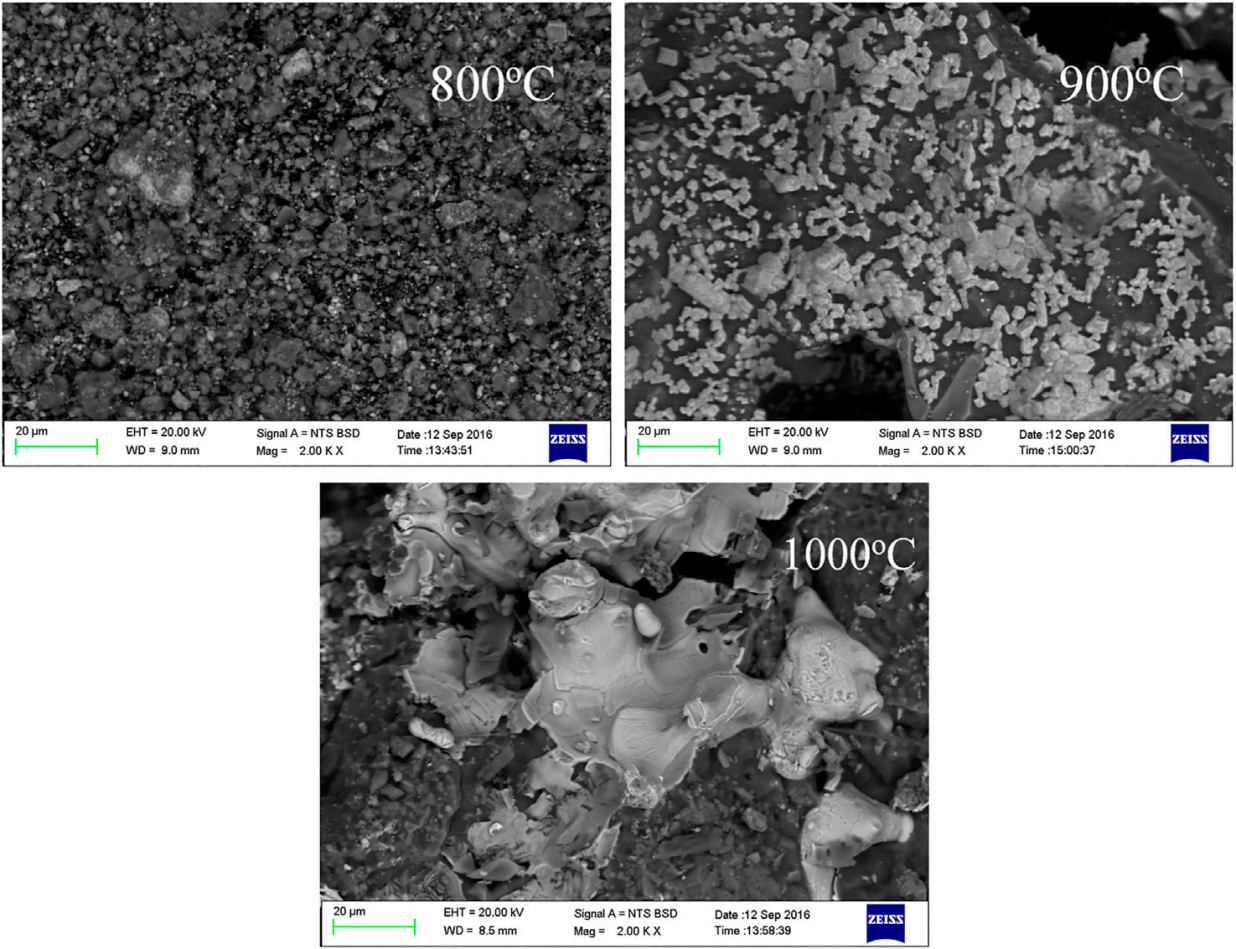
SEM images of roasted laterite ores with Na_2_S_2_O_3_ under different roasting temperatures.

Figure 9 presents the micrographs of the reduced PO at different temperatures for 90 min in the presence of sodium thiosulfate. The metallic particle grains of the nickel laterite grew larger at higher temperatures. Moreover, the metallic particle grains of the nickel laterite with sodium thiosulfate were much larger than those without sodium thiosulfate, which was consistent with previous results (Ilyas and Koike, 2012). With the increase in the calcination temperature from 800 to 1000°C, the nickel-iron particles dispersed in the minerals gradually accumulated and grew, and finally formed a lamellar eutectic. This may be due to the low-melting-point eutectic of Fe-FeS formed in the reduction process with the elemental sulfur produced by the decomposition of Na_2_S_2_O_3_ (Mcamish and Johnston, 1976). However, Na_2_O, which was formed by the decomposition of Na_2_S_2_O_3_, replaced the nickel wrapped in magnesium-containing silicate minerals and formed low-melting-point substances (Na_2_SiO_3_) (Li et al., 2012). The low melting point eutectic could directionally aggregate the nickel-iron particles on the solid surface. Therefore, it was beneficial for the magnetic separation process of the Ni-Fe enrichment.

**FIGURE 10:**
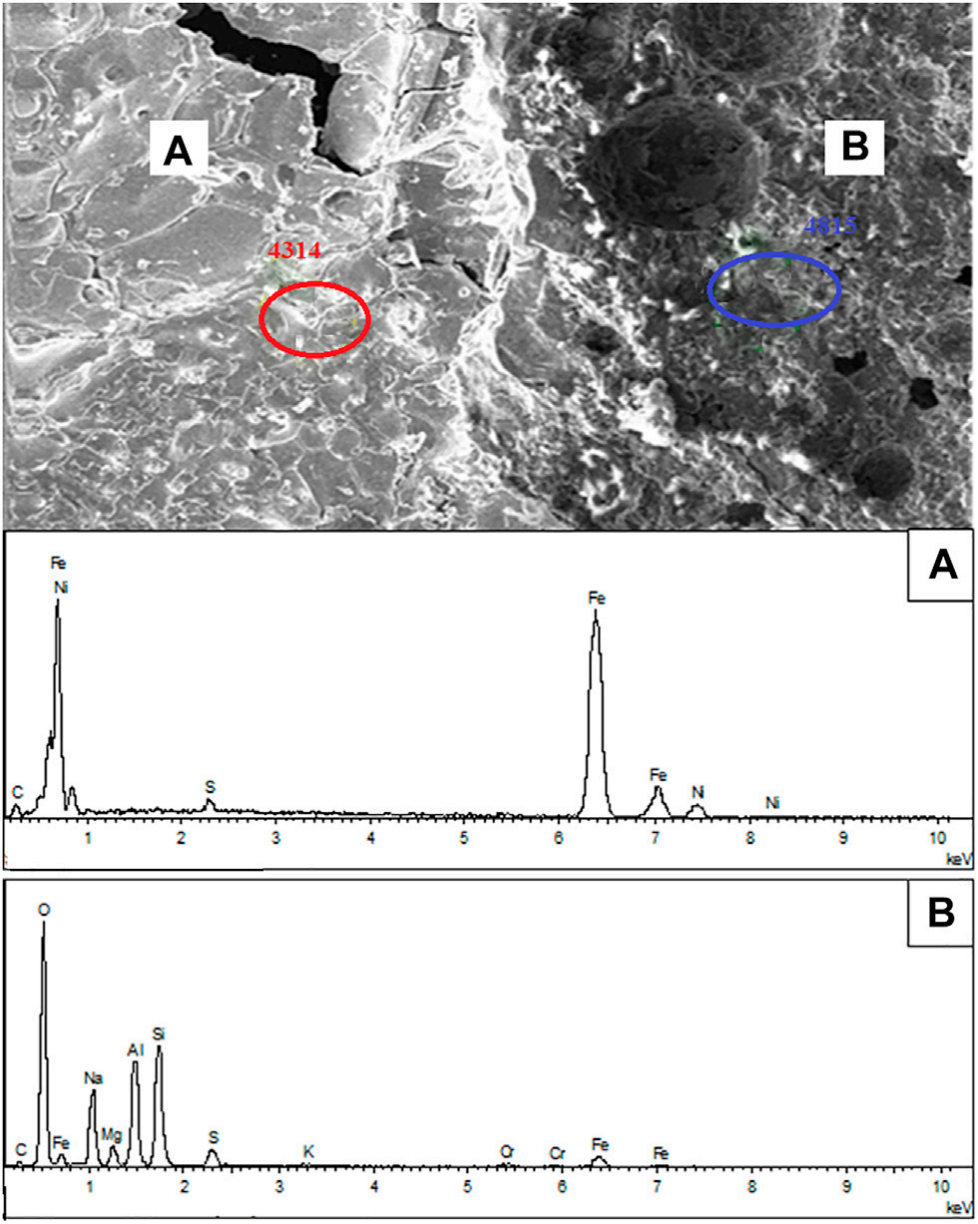
SEM-EDS analysis of the calcined product of laterite/Na_2_S_2_O_3_ blends: **(A)** bright area and **(B)** gray area.

To explore the inherent nature of the aggregation and growth of nickel-iron particles, the reduction residue under 1,000°C with 20 wt% Na_2_S_2_O_3_ was analyzed by EDS, as shown in Figure 10. Two distinct zones-the bright area and the gray area-were evident on the surface. As indicated by the EDS result, the bright areas were mainly composed of Ni and Fe with small amount of S, which suggests the accumulation of nickel-iron particles. However, the gray areas mainly contained Si, Al, Na, Mg, O and small amounts of Fe, indicating the presence of gangue, such as magnesium and sodium silicate minerals.

The possible reasons for the Ni grade and recovery improvement by Na_2_S_2_O_3_ are as following. On the one hand, the Na_2_S_2_O_3_ could decompose to S, which could participate in the formation of Fe-FeS as the low point eutectic phase (Lu et al., 2013). With the help of this eutectic phase, the nickel-iron particles could be directionally accumulated and then benefit the subsequent magnetic separation progress. On the other hand, the Na_2_S_2_O_3_ could also provide Na_2_O (alkali metal salt), which could replace the nickel wrapped in magnesium-containing silicate mineral. Therefore, the recovery rate of the nickel concentration was improved.

#### Mechanism of Sodium Thiosulfate in Promoting Reduction of Laterite Ore by H_2_

**FIGURE 11:**
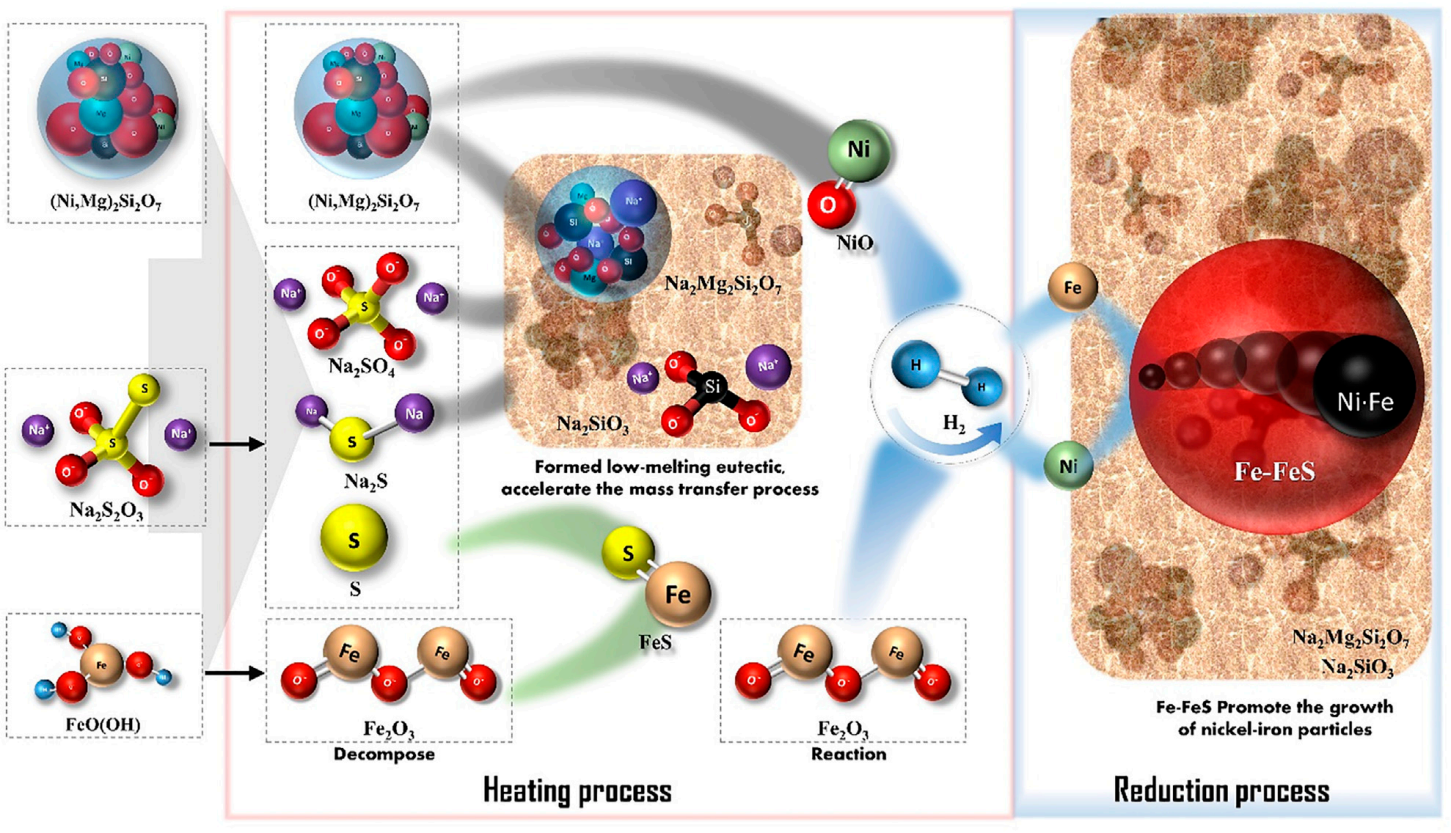
Hydrogen-thermal reduction mechanism of laterite ore enhanced by Na_2_S_2_O_3_.

The mechanism of Na_2_S_2_O_3_ for promoting reduction of the laterite ore is shown in Figure 11. Na_2_S_2_O_3_ played multiple role in the hydrogen reduction as sulfur and alkali metal salt providers. The reaction mechanism is described as follows: 1) During the heating step, Na_2_S_2_O_3_ decomposed to S, Na_2_SO_4_, and Na_2_S. 2) S then reacted with iron oxides, which decomposed from goethite in the laterite nickel ore to form FeS; Na_2_SO_4_ and Na_2_S destroyed the structures of the nickel-bearing silicate minerals at higher temperatures, releasing the nickel wrapped in magnesium-containing silicate minerals and forming a low-melting-point eutectic phase (Na_2_Mg_2_Si_2_O_8_). 3) During the H_2_ reduction stage, NiO and iron oxide were reduced to their metal phases, after which the fine nickel and iron particles directionally aggregated and grew with the help of FeS-Fe (low melting point eutectic phase). 4) As large amounts of sodium silicate entered the liquid phase, element migration was enhanced and hence the reduction and growth process of the Fe-Ni were accelerated.

## Conclusion

In summary, sodium thiosulfate was firstly applied to enhance the reduction of laterite ore by H_2_. The maximum values of 9.97 and 99.24% were achieved for the nickel grade and recovery, respectively, at 1,000°C with the addition of 20 wt% Na_2_S_2_O_3_. Na_2_S_2_O_3_ played multiple roles as sulfur and alkali metal salt providers simultaneously to improve the reduction. A possible mechanism of sodium thiosulfate in promoting the reduction of laterite ore can be concluded as follows: Na_2_S_2_O_3_ was firstly decomposed to S, Na_2_SO_4_, and Na_2_S. The alkali metal salts then destroyed the structures of nickel-bearing silicate minerals to liberate nickel as nickel oxide, while S reacted with Fe to form a low melting point eutectic phase of FeS-Fe. Fine nickel and iron particles were directionally aggregated and grew due to the capillary forces of the FeS-Fe system under H_2_ reduction. In addition, the massive formation of sodium silicate in the liquid phase reduced the reaction temperature by enhancing the element migration ability simultaneously. Hence, the reduction and growth process of the Fe-Ni were accelerated.

## Data Availability

The original contributions presented in the study are included in the article/Supplementary Material, further inquiries can be directed to the corresponding authors.
