# Supplementary material for: A Robust Recovery of Ni From Laterite Ore Promoted by Sodium Thiosulfate Through Hydrogen-Thermal Reduction
Source: Front Chem. 2021 Jun 25;9:704012. doi: 10.3389/fchem.2021.704012 (PMC8267239; doi:10.3389/fchem.2021.704012)
Supplement: Supplementary file 1 [file Table1.docx]

Supplementary Material

# Supplementary Data

**Table S1** Thermodynamic calculations data based on Equations (6)-(18) in the temperature range of 298-1500K.

| Temperature/K | ${\Delta G}_{T}^{ϴ}$/J | | | | | |
| --- | --- | --- | --- | --- | --- | --- |
|  | (6) | (7) | (8) | (9) | (10) | (11) |
| 298 | 15550 | -138336 | 141321 | 141322 | 154625 | 380111 |
| 300 | 15436 | -138258 | 140848 | 140848 | 154119 | 379161 |
| 400 | 12003 | -134317 | 117628 | 117628 | 129440 | 332363 |
| 500 | 8092 | -130950 | 950005 | 95001 | 105346 | 286942 |
| 600 | 3836 | -134431 | 746059 | 74606 | 83161 | 246581 |
| 700 | -770 | -140032 | 551170 | 55117 | 61765 | 208420 |
| 800 | -5733 | -146867 | 361675 | 36168 | 40853 | 171658 |
| 900 | -11063 | -154762 | 177127 | 17713 | 20407 | 136179 |
| 1000 | -17118 | -163760 | -246 | -247 | 369 | 101951 |
| 1100 | -25499 | -173995 | -17677 | -17677 | -19584 | 69008 |
| 1200 | -34403 | -186878 | -33673 | -33673 | -40045 | 39123 |
| 1300 | -43794 | -196711 | -48120 | -48121 | -60431 | 12487 |
| 1400 | -54263 | -207046 | -63698 | -63699 | -80440 | -13704 |
| 1500 | -67316 | -218253 | -81311 | -81312 | -100065 | -43075 |

**Table S2** Thermodynamic calculations data based on Equations of (6)-(18) in the temperature range of 298-1500K.

| Temperature/K | ${\Delta G}_{T}^{ϴ}$/J | | | | |
| --- | --- | --- | --- | --- | --- |
|  | (12) | (13) | (14) | (15) | (16) |
| 298 | 551621 | -16989 | 55918 | 170884 | 54862 |
| 300 | 550197 | -17088 | 55905 | 170685 | 54732 |
| 400 | 480008 | -21879 | 52738 | 161264 | 48794 |
| 500 | 411516 | -26246 | 49364 | 152044 | 43375 |
| 600 | 350082 | -30186 | 45796 | 141945 | 37915 |
| 700 | 291544 | -33929 | 42030 | 131368 | 32614 |
| 800 | 234749 | -37500 | 38203 | 120571 | 27517 |
| 900 | 149543 | -40903 | 34191 | 109285 | 22519 |
| 1000 | 125897 | -44164 | 29991 | 97482 | 17757 |
| 1100 | 73877 | -47308 | 25657 | 85243 | 13247 |
| 1200 | 26159 | -50353 | 21136 | 72457 | 8759 |
| 1300 | -16962 | -53315 | 12536 | 59116 | 4586 |
| 1400 | -59239 | -56210 | 6177 | 44963 | 733 |
| 1500 | -100813 | -59408 | 2683 | 29115 | -2788 |
